# Surgical resection should be taken into consideration for the treatment of small gastric gastrointestinal stromal tumors

**DOI:** 10.1186/1477-7819-11-273

**Published:** 2013-10-13

**Authors:** Jianjun Yang, Fan Feng, Mengbin Li, Li Sun, Liu Hong, Lei Cai, Wenbin Wang, Guanghui Xu, Hongwei Zhang

**Affiliations:** 1 Department of Digestive Surgery, Xijing Hospital of Digestive Diseases, the Fourth Military Medical University, 127 West Changle Road, 710032, Xi’an, Shaanxi, China

**Keywords:** Gastric gastrointestinal stromal tumor, Malignant potential, Mitotic index, Gene mutation

## Abstract

**Background:**

The National Comprehensive Cancer Network (NCCN) recommends conservative follow-up for gastric gastrointestinal stromal tumors (GISTs) less than 2 cm. The aim of the present study was to investigate the clinical and pathological features of small gastric GISTs, re-evaluate the risk potential, and discuss the treatment strategy of small gastric GISTs.

**Methods:**

In this retrospective study, 63 cases of small gastric GISTs (less than 2 cm) were resected surgically from May 2010 to March 2013 in our department. Clinicopathological factors were collected and the malignant potential of small gastric GISTs was analyzed.

**Results:**

The mitotic index of 14 out of 63 cases (22.22%) exceeded 5. The malignant potential of small gastric GISTs was related to tumor location (*P* = 0.0218). The mitotic index of 4 out of 8 GISTs (50%) located in gastric cardia exceeded 5, 8 out 28 GISTs (28.57%) located in the gastric fundus exceeded 5, and only 2 out of 27 GISTs (7.41%) located in the gastric body exceeded 5. We also discovered a good consistency between mitotic index and Ki-67 expression of small gastric GISTs.

**Conclusions:**

Gastric GISTs less than 2 cm also have malignant potential. Thus, we recommended surgical resection of all small gastric GISTs once diagnosed.

## Background

Gastrointestinal stromal tumors (GISTs) are the most common mesenchymal tumor of the gastrointestinal tract and represent 1% to 2% of all gastrointestinal malignancies [1]. They are considered to be derived from the interstitial cells of Cajal, the pacemaker cells of the gastrointestinal tract [2]. This has been established by immunohistochemical staining of GISTs for CD117, CD34, smooth muscle actin, desmin and S-100 [3]. In 1998, Hirota *et al*. reported that GISTs are associated with gain-of-function mutations in the *KIT* proto-oncogene [2]. Histologically, most GISTs display spindle cell morphology (70%), whereas a minority is of epithelioid (20%) or mixed phenotypes (10%) [4]. GISTs can occur anywhere throughout the gastrointestinal tract and are seen most commonly in the stomach (40 to 70%), small intestine (20 to 40%), and colon and rectum (5 to 15%) [5]. Rare cases have been reported in the esophagus, appendix, greater omentum, and gallbladder [6]. Patients with gastric GISTs may be completely asymptomatic or present with abdominal pain, dyspepsia, anorexia, bleeding, obstruction or tarry stool [7].

According to the NCCN guideline [8], gastric GISTs less than 2 cm and with a mitotic index (number of mitoses per 50 HPF (high-power fields)) less than 5 were considered as very low risk. Thus, surgical intervention with negative margins is the treatment of choice for primary, localized gastric GISTs larger than 2 cm, while conservative follow-up is suggested for lesions less than 2 cm [9-11]. However, it is believed that all GISTs have malignant potential [12], including small gastric GISTs (less than 2 cm). To date, little is known about the natural course of small gastric GISTs, and no literature has reported the mitotic index and gene mutation spectrum of small gastric GISTs.

Given this situation, we presume that the treatment principle of gastric GISTs less than 2 cm should be reconsidered. In the present study, we retrospectively analyzed the clinical and pathological data of 63 patients with gastric GISTs less than 2 cm. The aim of the present study was to reevaluate the risk potential and reconsider the treatment principle of small gastric GISTs.

## Methods

### Patients

This study was performed in the Xijing Hospital of Digestive Diseases affiliated to the Fourth Military Medical University. From May 2010 to March 2013, a total of 63 patients who were suspected of having a small gastric GIST (maximum diameter ≤2 cm) as a result of examination by endoscopic ultrasound (EUS) and enhanced abdominal computed tomography (CT) were enrolled in the present study. Surgical resection was performed by surgeons who are specialized in gastric surgery in our department. This study was approved by the Ethics Committee of Xijing Hospital, and written informed consent was obtained from all patients before surgery.

### Pathology

All the specimens were fixed in 10% neutral formalin immediately after resection and embedded routinely for histologic examination in the Pathology Department in the Xijing Hospital. Immunohistochemistry was performed on 3-μm sections according to the manufacturer's instructions and the following antibodies: CD117 (polyclonal, 1:200; DAKO, Hamburg, Germany), CD34 (clone QBEnd10, 1:200; Immunotech, Hamburg, Germany), Discovered on GIST-1 (monoclonal DOG-1, 1:200; Novocastra, Newcastle, UK), Ki67 (clone MIB1, 1:150, DAKO). Histological type (spindle, epithelioid, mixed) and mitotic index were also detected by hematoxylin and eosin stain.

### Gene mutation detection

DNA of the GIST tissues was isolated using a QIAmp DNA FFPE Tissue kit according to manufacturer’s instructions (Qiagen, Hilden, Germany). Polymerase chain reaction (PCR) was used to amplify *KIT* exons 9, 11, 13 and 17 and *PDGFRA* exons 12 and 18. The PCR reaction was performed using a Taq PCR Master Mix according to manufacturer’s instructions (Qiagen, Hilden, Germany). Mutations were confirmed by comparing the sequencing results with gene sequences in the NCBI Genbank. Primers used in PCR were listed as follows: *KIT* exon 9 forward: TCCTAGAGTAGTAAGCCAGGGCTT, *KIT* exon 9 reverse: TGGTAGACAGAGCCTAAACATCC, *KIT* exon 11 forward: CCAGAGTGCTCTATAGACTG, *KIT* exon 11 reverse: AGCCCCTGTTTCATACTGAC, *KIT* exon 13 forward: GACATCAGTTTGTCAGTTG, *KIT* exon 13 reverse: GCAAGAGAGAACAACAG, *KIT* exon 17 forward: GTGAACATCATTCAAGGCG, *KIT* exon 17 reverse: TTACATTATGAAAGTCACAGG, *PDGFRA* exon 12 forward: TCCAGTCACTGTCCTGCTTC, *PDGFRA* exon 12 reverse: GCAAGGGAAAAGGGAGTCTT, *PDGFRA* exon 18 forward: ACCATGGATCAGCCAGTCTT, *PDGFRA* exon 18 reverse: TGAAGGAGGATGAGCCTGACC.

### Statistical analysis

Data were processed using SPSS 16.0 for Windows (SPSS Inc., Chicago, IL, USA). Numerical variables were expressed as the mean ± SD unless otherwise stated. Discrete variables were analyzed using the Chi-square test or Fisher's exact test. The consistency of the mitotic index and the Ki-67 was analyzed using McNemar’s test and the Kappa test. The *P* values were considered to be statistically significant at the 5% level.

## Results

### Patient and tumor characteristics

Clinical and pathological characteristics of the patients and tumors are summarized in Table 1. Sixty-three patients meeting the criteria for the diagnosis of EUS-suspected small gastric GISTs were enrolled, and comprised 39 men and 24 women. The average age was 62.44 ± 9.76 years (range: from 39 to 89 years). Approximately 73.02% of the patients were symptomatic. Presenting symptoms included abdominal pain (28.57%), bleeding (7.94%), and abdominal discomfort (36.51%). On the basis of the EUS, pathology and operative report, 8 tumors were located in the cardia, 28 tumors in the gastric fundus, and 27 tumors in the gastric body. Approximately 22.22% of the patients had more than 5 mitotic figures per 50 HPF, and 22.22% of the patients had a positive Ki-67 stain (>5%). A total of 61 of the 63 cases studied (96.83%) stained positive for CD117, 61 of the 63 cases (96.83%) stained positive for DOG-1, and 62 of the 63 cases (98.41%) stained positive for CD34. Molecular analysis revealed *KIT* exon 11 mutation in 47 cases, *KIT* exon 9 mutation in 3 cases, *KIT* exon 13 mutation in one case, *KIT* exon 17 mutation in one case, *PDGFRA* exon 18 mutation in one case, and wild type in 10 cases.

**Table 1:** **Clinical and pathological features of small gastric gastrointestinal stromal tumors (**GISTs**)**

**Characteristics**	**Cases (n = 63)**
Age	62.44 ± 9.76
Sex	
Male	39
Female	24
Tumor location	
Cardia	8
Gastric fundus	28
Gastric body	27
Clinical symptoms	
Pain	18
Bleeding	5
Discomfort	23
Asymptomatic	17
Mitotic index	
≤5	49
>5	14
Ki-67	
≤5	49
>5	14
CD117	
Positive	61
Negative	2
DOG-1	
Positive	61
Negative	2
CD34	
Positive	62
Negative	1
Gene mutation	
*KIT* exon 11	47
*KIT* exon 9	3
*KIT* exon 13	1
*KIT* exon 17	1
*PDGFRA* exon 12	0
*PDGFRA* exon 18	1
Wild type	10

### The relationship between mitotic activity and clinical features

The correlations of mitotic activity and clinical features are summarized in Table 2. The results show that the mitotic index was not statistically different and had no correlations with age, sex, tumor size and clinical symptoms. However, the mitotic index was related to the location of gastric GISTs (*P* <0.05). The ratio of mitotic index (>5 per 50 HPF) was highest in the GISTs located in the cardia (50%) and lowest in the gastric body (8%). No GIST was found in the gastric antrum in our present study. Although the ratio of mitotic index (>5 per 50 HPF) of gastric GISTs between 1 to 2 cm was higher than that of gastric GISTs less than 1 cm (33.33% versus 12.12%), there was no significant difference between the two groups.

**Table 2:** The relationship between mitotic activity and clinical features

**Characteristics**	**Mitotic index**		**Statistics**
Age	≤5	>5	
≤50	7	1	*P* = 0.1400
51to 60	8	4	
61to 70	27	4	
>70	7	5	
Sex			
Male	31	8	*P* = 0.7591
Female	18	6	
Tumor size			
≤1 cm	29	4	*P* = 0.0678
1 to 2 cm	20	10	
Tumor location			
Cardia	4	4	*P* = 0.0218
Gastric fundus	20	8	
Gastric body	25	2	
Clinical symptoms			
Symptomatic	34	12	*P* = 0.3155
Asymptomatic	15	2	

McNemar’s test and the Kappa test were used to measure the agreement between mitotic index and expression of Ki-67 of small gastric GISTs. The results in Table 3 show a good consistency between mitotic index and Ki-67 expression (*P* = 1.0000, Kappa = 0.724).

**Table 3:** **The consistency of mitotic index and Ki-67 expression of small gastric gastrointestinal stromal tumors (**GISTs**)**

		**Ki-67**		**Statistics**
		**≤5**	**>5**	
Mitotic index	≤5	46	3	*P* = 1.0000
	>5	3	11	Kappa = 0.724

## Discussion

The management of GISTs is generally based on tumor size because biopsy is not recommended and mitotic index cannot be easily and accurately determined [13]. The National Comprehensive Cancer Network recommends surgical resection for tumors greater than 2 cm because of malignant potential, and lesions less than 2 cm can be conservatively followed up [14]. As a result, the malignant potential of small gastric GISTs could not be accurately determined due to the lack of mitotic index based on pathology. However, every GIST is now regarded as potentially malignant, and even GISTs with low mitotic rates were reported to recur locally or to metastasize [15]. The present study sought to identify the clinical and pathological features of small gastric GISTs and to discuss the treatment strategy of small gastric GSITs.

As a specific marker of GISTs, CD117 has good sensitivity and was highly expressed in nearly 85% to 94% of cases [16]. The high sensitivity and specificity of CD117 is a useful marker in differentiating GIST from other mesenchymal tumors of the gastrointestinal tract. DOG1 (Discovered on GIST-1) is a newly identified marker of GISTs, West *et al*. reported ubiquitous expression of DOG-1 in GISTs and demonstrated the immunoreactivity for DOG-1 in 97.8% of GISTs [17]. Many reports showed that the sensitivity for CD117 and DOG1 are almost the same, and the two factors have consistency. As a hematopoietic progenitor cell antigen, CD34 is commonly present in GISTs but is less specific than CD117 and DOG1. The positive rate of CD34 is approximately 60% to 70% [18]. In our present study, the clinical and pathological characteristics of small gastric GISTs were in agreement with the references reported. These indicate that there is no significant difference in the clinical and pathological features between the small gastric GISTs in our study and the GISTs reported previously.

Tumor size and mitotic index are the best prognostic indicators for determining the malignant potential of GISTs [19]. In our present study, although all the gastric GISTs were less than 2 cm, the mitotic index of 14 small gastric GISTs was greater than 5 per 50 HPF. It was striking to observe that 22.22% of small gastric GISTs showed low risk, which indicated the malignant potential and implied the necessity of surgical resection of small gastric GISTs. Furthermore, we analyzed the relationship between tumor size (≤1 cm versus 1 to 2 cm) and mitotic index. We found that there was no significant difference between the two groups, and the mitotic index of 4 out of 33 gastric GISTs (≤1 cm) was greater than 5 per 50 HPF. In this situation, we think that all GISTs should be resected once diagnosed. Besides tumor size and mitotic index, the location of GIST is also considered as one of the risk factors. It is reported that the location of GISTs in the gastric cardia and gastroesophageal junction is an unfavorable prognostic factor [20]. In our present study, 8 gastric GISTs were located in the gastric cardia, and the mitotic index of 4 cases exceeded 5, demonstrating that GISTs located in the gastric cardia possess more malignant potential than those located in the gastric fundus and gastric body.

The current management policy for gastric GISTs less than 2 cm is usually conservative, unless tumors grow or symptoms occur [21]. In our present study, 46 of 63 cases (73.02%) were presented with symptoms including pain, bleeding and discomfort. The high rate of presenting symptoms resulted from the combination of gastric cancer and gastric GIST of patients in our study. Even in the remaining 17 asymptomatic patients, the mitotic index of 2 cases was more than 5 per 50 HPF, indicating malignant potential. These findings also indicate that gastric GISTs less than 2 cm should be resected once diagnosed because most of the small gastric GISTs presented with symptoms, and some asymptomatic cases possessed malignant potential.

In 1998, Hirota *et al*. reported their groundbreaking discovery of *KIT* mutations in GISTs. It is now established that 70% to 80% of GISTs harbor a *KIT* gene mutation [22]. Most of these are exon 11 mutations, which cause constitutively activated receptors leading to unregulated autophosphorylation of the intracytoplasmic tyrosine kinases [23]. *KIT* mutations in exons 9, 13 and 17 are less common and have been associated with more aggressive tumor behavior [20]. *PDGFRA* mutations occur in approximately 20% to 25% of gastric GISTs, and most commonly in exon 18 [24]. *KIT* and *PDGFRA* mutations are mutually exclusive [25]. Very rare cases may have mutations in the BRAF kinase [26]. GISTs without a mutation in either *KIT* or *PDGFRA* genes account for about 10% to 15% of GISTs and are known as wild type [27]. In our present study, 74.60% of small gastric GISTs harbor a *KIT* exon 11 mutation, 4 cases (4.76%) harbor a *KIT* exon 9 mutation, one case (1.59%) harbors a *KIT* exon 13 mutation and one case (1.59%) harbors a *KIT* exon 17 mutation. One case (1.59%) harbors a *PDGFRA* exon 18 mutation, and 10 cases (15.87%) were wild type. These results demonstrate that the gene mutation spectrum of small gastric GISTs in our present study is in agreement with the references reported.

Some authors have proposed the use of Ki-67 as a more objective parameter for risk assessment, because multivariate analyses in several studies do indicate that Ki-67 index could be independently used as an outcome predictor [28]. In our present study, the consistency of the mitotic index and Ki-67 expression was analyzed using McNemar’s test and the Kappa test. The results showed a good consistency between mitotic index and Ki-67 expression. This indicated that Ki-67 expression may also be considered as a prognostic indicator for determining the malignant potential of gastric GISTs.

There are several limitations in the present study. First, no recurrence-free survival rate of patients who received surgical resection of small gastric GISTs could be obtained. Second, further studies should be carried out to investigate the necessity of medication after surgical resection. Third, multicenter randomized controlled studies should be carried out to confirm the benefit of surgical resection of small gastric GISTs compared with conservative patients.

## Conclusions

Through pathological examination and gene mutation analysis, we found that some gastric GISTs less than 2 cm also harbor malignant potential, and recommend surgical resection of all small gastric GISTs once diagnosed. Thus, the treatment principle of gastric GISTs less than 2 cm should be reconsidered.

## Abbreviations

CT: Enhanced abdominal computed tomography; DOG1: Discovered on GIST-1; EUS: Endoscopic ultrasound; GIST: Gastrointestinal stromal tumor; HPF: High-power fields; NCCN: National Comprehensive Cancer Network.

## Competing interests

The authors declared that they have no competing interests.

## Authors’ contributions

YJJ designed the study; FF participated in data analysis; LMB carried out the operation; SL participated in acquisition of data; HL participated in immunohistochemisty; CL participated in gene mutation detection; WWB helped to draft the manuscript; XGH performed the statistical analysis; and ZHW, as director of the department, coordinated its execution and design, and drafted and produced the final version of the manuscript. All authors reviewed and approved the final manuscript.
